# Using single-nucleus RNA-sequencing to interrogate transcriptomic profiles of archived human pancreatic islets

**DOI:** 10.1186/s13073-021-00941-8

**Published:** 2021-08-10

**Authors:** Giorgio Basile, Sevim Kahraman, Ercument Dirice, Hui Pan, Jonathan M. Dreyfuss, Rohit N. Kulkarni

**Affiliations:** 1 Section of Islet Cell and Regenerative Biology, Joslin Diabetes Center and Harvard Medical School, Boston, MA 02215 USA; 2 Current Address: Department of Pharmacology, New York Medical College School of Medicine, Valhalla, NY 10595 USA; 3 Bioinformatics and Biostatistics Core, Joslin Diabetes Center and Harvard Medical School, Boston, MA USA; 4 Department of Medicine, Brigham and Women’s Hospital, Boston, MA USA; 5 Harvard Stem Cell Institute, Harvard Medical School, Boston, MA USA

**Keywords:** Transplanted human islets, Single-nucleus RNA-sequencing, Single-cell RNA-sequencing, Human β-cells, Frozen samples, Archived tissue, Diabetes

## Abstract

**Background:**

Human pancreatic islets are a central focus of research in metabolic studies. Transcriptomics is frequently used to interrogate alterations in cultured human islet cells using single-cell RNA-sequencing (scRNA-seq). We introduce single-nucleus RNA-sequencing (snRNA-seq) as an alternative approach for investigating transplanted human islets.

**Methods:**

The Nuclei EZ protocol was used to obtain nuclear preparations from fresh and frozen human islet cells. Such preparations were first used to generate snRNA-seq datasets and compared to scRNA-seq output obtained from cells from the same donor. Finally, we employed snRNA-seq to obtain the transcriptomic profile of archived human islets engrafted in immunodeficient animals.

**Results:**

We observed virtually complete concordance in identifying cell types and gene proportions as well as a strong association of global and islet cell type gene signatures between scRNA-seq and snRNA-seq applied to fresh and frozen cultured or transplanted human islet samples.

**Conclusions:**

We propose snRNA-seq as a reliable strategy to probe transcriptomic profiles of freshly harvested or frozen sources of transplanted human islet cells especially when scRNA-seq is not ideal.

**Supplementary Information:**

The online version contains supplementary material available at 10.1186/s13073-021-00941-8.

## Background

Type 1 diabetes (T1D) and type 2 diabetes (T2D) are both characterized by a progressive reduction of functional mass of insulin-producing β-cells [1, 2]. Therefore, restoring physiological numbers of endogenous β-cells, improving β-cell functionality, or generating insulin-producing β-like cells derived from stem cells for transplantation are promising strategies to resolve diabetes in patients. While the identification of factors able to stimulate β-cell proliferation [3–11] and the improvement of differentiation protocols to generate functional β-like cells [12–17] continue to evolve, gaining insights into dynamic changes in the global transcriptome of β-cells, especially following manipulation in an in vivo environment (e.g., in transplanted islets in humanized mouse models) is worth exploring.

One challenge when investigating the biology of pancreatic islets is the presence of at least five different hormone-secreting endocrine cell types and non-endocrine cells such as endothelial cells, glia, fibroblasts, pericytes, and immune cells (tissue-resident macrophages, mast cells, B cells, cytotoxic T cells) [18]. A mixture of such diverse cell types can hinder precise identification of cell-specific transcriptomes or exclusive biological signals when bulk tissue analyses are performed. To circumvent this limitation, over the past few years, multiple groups have utilized single-cell RNA-seq (scRNA-seq) methodologies on islet cells isolated from mouse [19, 20] or human pancreas [21–24]. These studies have focused on dissecting the transcriptomic signature of endocrine cell types across different ages [25] and to define differentially expressed genes in type 2 diabetic β-cells [26–29]. However, single-cell preparations are also known to have limitations, including the need to harvest live cells [30] which may inadvertently induce stress responses [31, 32]. These aspects gain relevance when identifying disease-related transcriptomic signatures in tissues obtained from multiple human donors at different time points that require immediate, although usually varied, processing and individual analyses.

To overcome these limitations [31], single-nucleus RNA-sequencing (snRNA-seq) has been employed in studies on tissues composed of diverse cell types, including the brain [33, 34], kidney [35–37], heart [38], skeletal muscle [39, 40], stria vascularis [41], retina [42], liver [43, 44], lung [45], or white [46, 47] and brown adipose tissue [48, 49] obtained from mice or human donors. Here, we report a side-by-side comparison between snRNA-seq and scRNA-seq to validate the robustness of the former as an alternative sequencing strategy. We propose that snRNA-seq is a reliable approach to interrogate the transcriptomic profiles of archived frozen tissues that, to our knowledge, has not been applied previously to pancreatic islets or engrafted tissues.

## Methods

### Animal studies

Healthy female 8–12-week-old non-obese diabetic (NOD)/severe combined immunodeficiency (SCID)-ɣ (NSG) mice were used as human islet transplant recipients. We used 4 animals in total, where a single animal received human islet preparation from a single donor. Animals were housed in the Animal Care Facilities at Joslin Diabetes Center on a 12-h light/12-h dark cycle with water and food ad libitum. Studies and protocols were approved by the Institutional Animal Care and Use Committee of the Joslin Diabetes Center (IACUC #05-01).

### Human islet studies

Human islets were obtained from 5 non-diabetic brain-dead donors. Islet preparations were generated by the Integrated Islet Distribution Program or Prodo laboratories according to the standard procedures [50] (Additional file 1: Table S1). All studies and protocols used were approved by the Joslin Diabetes Center’s Committee on Human Studies (CHS#5-05). Upon receipt, human islets were centrifuged, washed, transferred to Petri dishes (2000–3000 human islets equivalents [IEQs]/plate), and cultured with fresh Miami Media #1A (Cellgro) overnight at 37 °C and 5% CO_2_. Human islets from one donor (*N* = 1) were processed to isolate nuclei and obtain single-cell preparations as a common source for the scRNA-seq and snRNA-seq procedures (detailed below), while human islets from 4 donors (*N* = 4) were used in the kidney capsule transplantation experiments (detailed below).

### Human islet transplantations

On the day of the experiment, 1000 hand-picked IEQs from 4 separate human islet donors were transplanted under the kidney capsule of 8-to-12-week-old male NSG mice and the animals were followed for 4 weeks. At the end of the follow-up period, mice were sacrificed by cervical dislocation. Human islet grafts were rapidly dissected under the microscope, snap-frozen, and stored at − 80 °C.

### Single-nucleus isolation

Frozen or freshly cultured or engrafted human islet samples were transferred to Dounce tissue grinder tubes (D8938; Sigma) containing 0.5 ml ice-cold Nuclei EZ lysis buffer (NUC-101; Sigma) and homogenized with pestles A and B for 1 min each on ice. Samples were transferred to clean 15 ml tubes and homogenizers were rinsed with 1.5 ml buffer followed by 2 ml buffer and transferred to the same 15 ml tubes to obtain a final volume of 4 ml. The tubes were vortexed briefly at moderate speed and kept on ice for 5 min for cell lysis. To separate the nucleus and cytoplasm, the tubes were centrifuged at 500×*g* for 5 min at 4 °C. Supernatants containing cytoplasmic components were saved for later analyses. The pellet containing nuclei was resuspended in 0.5 ml cold buffer by vortexing briefly at moderate speed followed by the addition of 3.5 ml cold buffer. The nuclear suspension was mixed by vortexing briefly and set on ice for 5 min. The tubes were centrifuged at 500×*g* for 5 min, the supernatant was saved for later analysis, and the pellet was resuspended in suspension buffer (0.5 ml PBS containing 0.01% non-acetylated bovine serum albumin (BSA); Sigma and 0.1% RNase inhibitor; 2313A from Clontech). The nuclear suspension was pipetted ten times with a 1 ml tip, filtered through a 30-μm pre-separation filter (130-041-407; Miltenyi Biotech); cell number and cell viability were determined by cell counter using 0.4% trypan blue stain. The average number of total nuclei obtained from one-half graft was approximately 8.5 × 10^5^ nuclei (1.7 × 10^6^ cells/ml) with 5–10 μm size and 93.3 ± 1.1% dead cell rate (*n* = 32 samples across three independent experiments). The number of isolated nuclei was adjusted to 1000 nuclei/μl with suspension buffer and 10,000 nuclei were immediately used for the generation of Gel Beads In-Emulsion (GEMs) and barcoding. Leftover nuclei were saved for subsequent analysis.

### Human islet dispersion

Human islets were dissociated into single cells by TrypLE (12604-013; Thermo). Briefly, 1 ml TrypLE was added to human islet pellets and incubated at 37 °C for 12 min by mixing the tube every 3–4 min. At the end of incubation, TrypLE was neutralized by adding 9 ml cold Dulbecco’s modified Eagle’s medium with high glucose (DMEM HG, MT 10-017-CV; Corning) containing 10% fetal bovine serum (FBS) (10437028; Gibco). Cells were centrifuged at 1200 rpm for 3 min at 4 °C, supernatant was removed, and cells were resuspended in 0.5 ml Dulbecco’s phosphate-buffered saline (DPBS) (14190250; Gibco). The cell suspension was filtered through 30 μm filter to remove aggregates and counted. A final concentration of 1 × 10^6^ cells/ml cells was used for DAPI/Phase microscopy. For single-cell RNA-seq procedures, human islet cell suspensions were filtered using a 30-μm filter to remove any aggregates, and dead cells were excluded using Dead Cell Removal Kit (Miltenyi Biotec). Cells were counted and 10,000 cells were used for the generation of GEMs.

### DAPI/Phase microscopy

Fifty thousand dispersed human islet cells (50 μl) or isolated nuclei from frozen grafts were mixed with 50 μl of 4′,6-diamidino-2-phenylindole (double-stranded DNA staining, DAPI) solution (D9564; Sigma) diluted 1000 times in DPBS. Stained cells (10 μl) were loaded onto a Hemocytometer slide and imaged under a fluorescent microscope. Digital images were taken at 40× magnification with AXIO Imager A2 upright microscope equipped with X-Cite series 120Q light source, Axiocam 512 color camera. Bright field and fluorescent images were overlaid using ImageJ Software to determine complete cell lysis and nuclear integrity in isolated nuclear samples.

### Immunohistochemistry

Paraffin-embedded human islet graft sections were immunostained using anti-insulin (1:400, ab7842; abcam), anti-glucagon (1: 10,000, ab92517; abcam), and anti-somatostatin (1:500, ab30788; abcam) antibodies using previously described techniques [6, 51, 52]. Nuclei were labeled using DAPI. Images were acquired using a Zeiss LSM-710 Confocal Microscope and the Zen Black software (Carl Zeiss).

### Western blotting

Nuclei or cell pellets were lysed in RIPA buffer (pH 7.4) containing 100 mM NaF, 50 mM Hepes, 150 mM NaCl, 10% Glycerol, 1.2% Triton X, 1 mM MgCl_2_, 1 mM EDTA, 1 mM Na_3_VO_4_, protease inhibitor cocktail (P8340; Sigma), phosphatase inhibitor 2 (P5726; Sigma), and phosphatase inhibitor 3 (P0044; Sigma). The supernatants collected after the first and second centrifuge steps of nuclear isolation were used after adding protease and phosphatase inhibitors. Total protein concentration was determined by Pierce BCA Protein Assay Kit (23225; Thermo). Lysates (50 μg protein) were run in 8% SDS-PAGE and transferred to PVDF membrane (Millipore). Membranes were blocked for 10 min at room temperature with 5% milk and were incubated overnight at 4 °C with antibodies against Lamin A/C (1:1000, 4777; Cell Signaling Technology) or GAPDH (1:1000, 5174; Cell Signaling Technology). After three washes (10 min), the membranes were incubated for 1 h at RT with antibodies against rabbit IgG-HRP conjugate (1:1000, 170-6515: Bio-Rad) or mouse IgG-HRP conjugate (1:1000, 170-6516, Bio-Rad). After three 10-min washes, signals were visualized via Pierce ECL Western blotting substrate (PI32106; Thermo).

### RNA extraction and analysis

Cells were lysed in RLT buffer and RNA was extracted using the Qiagen RNeasy kit according to the manufacturer’s instructions. RNA concentrations were measured by Nanodrop (Thermo). RNA integrity was determined by using Agilent RNA 6000 Nano Kit (5067-1511; Agilent) according to the manufacturer’s instructions (Joslin Genomics Core).

### Single-nucleus and single-cell RNA-sequencing procedures

Gel Bead In-Emulsion (GEMs) were generated using the Chromium 3’ Single Cell Library Kit (v2, 10X Genomics, CA) according to the manufacturer’s instructions and adapting the adjustments for the cDNA and libraries amplification steps, as recommended by 10X genomics for snRNA-seq procedures. Briefly, 10,000 cells or nuclei were combined with Single Cell Master Mix and encapsulated into the barcoded Gel Beads through the Chromium™ Controller. After GEM-RT incubation, cDNA samples were recovered, purified, and amplified through a cDNA Amplification Reaction using a 14-cycle setting. Quality controls on the undiluted amplified cDNA samples were performed using a High Sensitivity DNA Kit (Agilent, CA) on a 2100 BioAnalizer (Agilent, CA) platform. Libraries were then constructed following Fragmentation and Adaptor Ligation. Sample Index PCR was performed adjusting the reactions at 15 cycles. Finally, purified libraries were run on 2100 BioAnalizer (Agilent, CA) using a High Sensitivity DNA Kit (Agilent, CA) to evaluate the quality of the ~ 400 bp fragments.

### Next generation sequencing

Single-nucleus and single-cell libraries were sent for sequencing at GeneWiz or NovoGene laboratories. Samples were run in independent lanes on a HiSeq 4000 platform (Illumina, CA), using a coverage of 500,000 pair-ended reads targeted per cell.

### Single-nucleus and single-cell RNA-seq data analysis

Both the single-nucleus (snRNA-seq) and single-cell (scRNA-seq) RNA-sequencing datasets were produced using cultured human islet samples obtained from the same donor. In particular, we generated single-cell and single-nucleus libraries using 4 technical replicates for each method. Gene counts were obtained by Cell Ranger (10x Genomics, CA) using the human reference genome. To eliminate empty droplets and technical artifacts, we applied Cell Bender [53]. Cell Bender concludes that droplets are empty if the probability that they are empty > 50%, and then estimates ambient RNA among non-empty droplets. We normalized each sample’s data using sctransform [54], part of the Seurat toolkit, and detected and removed doublets with DoubletFinder [55]. To analyze the complete dataset, we combined the 4 samples, including genes that are detected in at least 3 cells and including cells where at least 200 genes are detected. From the combined dataset, we filtered out nuclei that have more than 20% mitochondrial unique molecular identifiers (UMI).

For the engrafted snRNA-seq, gene counts were generated by Cell Ranger (10x Genomics, CA) using the human-mouse joint reference genome. To eliminate empty droplets and technical artifacts, we applied Cell Bender [53]. We normalized each sample’s data using sctransform [54], part of the Seurat toolkit, and detected and removed doublets with DoubletFinder [55]. To analyze the complete dataset, we combined the 4 samples, including genes that are detected in at least 3 cells and including cells where at least 200 genes are detected. From the combined dataset, we filtered out nuclei that have more than 20% mitochondrial UMI and more than 25% mouse UMI and removed all mouse genes in the remaining nuclei. For each of them (i.e., the engrafted islet snRNA-seq, the scRNA-seq, or the snRNA-seq from cultured human islets), we normalized the dataset of combined samples with sctransform [54], part of the Seurat toolkit; and then using Seurat, we identified clusters and marker genes per cluster and plotted the data as Uniform Manifold Approximation and Projection (UMAP), heatmaps, and violin plots. For all datasets, genes were considered expressed in a cell or nucleus if they had at least one UMI. We evaluated gene expression levels between datasets per cell type by evaluating the linear regression of one dataset’s mean natural log of counts per gene against the other datasets for the same gene and reporting the *R*^2^, which is the square of the Pearson correlation coefficient, and the *p* value.

### Integration with published scRNA-seq datasets

The ability of Seurat [56] to integrate single**-**cell datasets was demonstrated on publicly-available human pancreatic islet scRNA-seq datasets spanning 27 donors, four laboratories, and five technologies: The datasets that have been used in this article have been deposited in the public Genomic Spatial Event Database (GSE) and in the ArrayExpress Database at the EMBL-EBI. For InDrop, accession number GSE84133 (https://www.ncbi.nlm.nih.gov/geo/query/acc.cgi?acc=GSE84133) [21]; for CelSeq2, accession number GSE85241 (https://www.ncbi.nlm.nih.gov/geo/query/acc.cgi?acc=GSE85241) [23]; for SMART-Seq2, accession number E-MTAB-5061 (https://www.ebi.ac.uk/arrayexpress/experiments/E-MTAB-5061/) [26]; for Fluidigm C1, accession number GSE86469 (https://www.ncbi.nlm.nih.gov/geo/query/acc.cgi?acc=GSE86469) [27]; and for CelSeq, accession number GSE81076 (https://www.ncbi.nlm.nih.gov/geo/query/acc.cgi?acc=GSE81076) [57] were used. We followed Seurat instructions to normalize these datasets with sctransform [54], identify integration anchors (we used dimensionality 25, consistent with the dimensionality we applied in Seurat analysis of our datasets), and construct our reference with Seurat function *IntegrateData*. We then used Seurat to project this universal scRNA-seq dataset, named “reference,” onto our datasets (i.e., the engrafted islet snRNA-seq and the scRNA-seq and snRNA-seq from cultured human islets) to harmonize the data.

## Results

### Isolation of single nuclei from frozen human islets

**Fig. 1:**
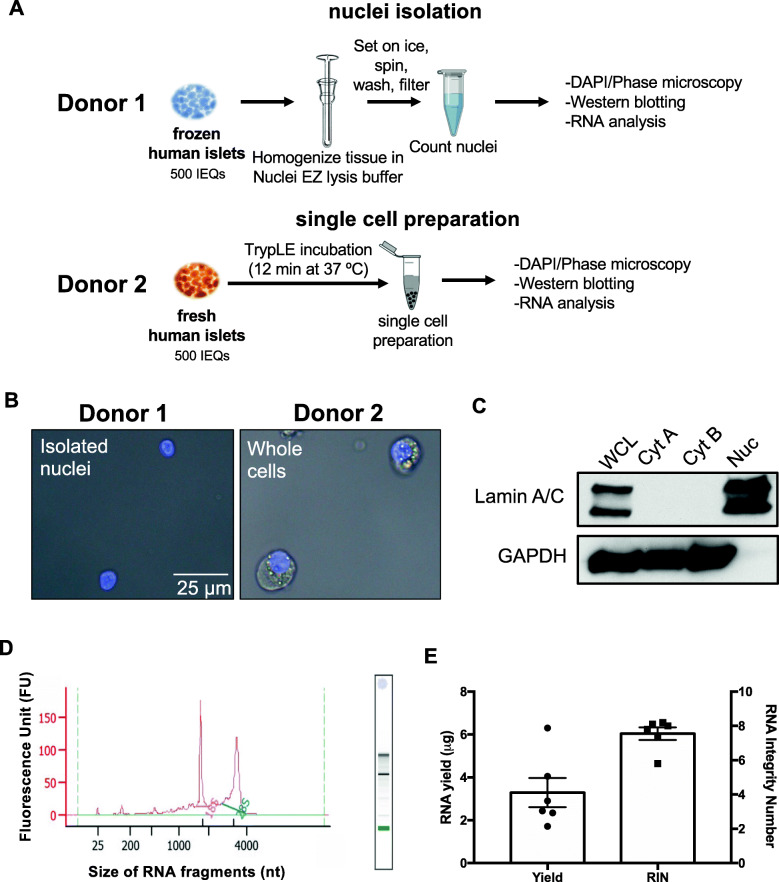
Single nuclei isolation from frozen human islet samples. **A** Experimental workflow of isolation of nuclei from frozen cultured human islets (donor 1) and human islet dispersion into single cells (donor 2). **B** Isolated nuclei from frozen human islets (donor 1) and dissociated human islet cells from fresh intact islets (donor 2, whole cells) were imaged by DAPI/Phase microscopy. Left image: Intact nuclei stained in blue without cytoplasm surrounding the isolated nucleus . Right image: Nuclei stained in blue surrounded by cytoplasm of human islet cells. Scale bar is 25 μm. **C** Western blot analysis of human pancreatic islet cells (whole cell lysate; WCL, obtained from donor 2), supernatant collected after the first centrifuge step (cytosolic fraction A; Cyt A) and second centrifuge step (cytosolic fraction B; Cyt B) of the nuclear isolation protocol, and isolated nuclei (nuclear fraction; Nuc, obtained from Donor 1). LaminA/C is a nuclear marker; GAPDH is a cytoplasmic marker. **D** RNA was isolated from islet cell nuclei and analyzed by Bioanalyzer. Representative electropherogram showing two major peaks (18S and 28S rRNA) indicates minimal degradation of total RNA. The *X*-axis indicates the size of RNA fragments (nt: nucleotides) and the *Y*-axis represents the intensity of the fluorescence signal (FU, fluorescence unit). **E** Yield (μg, left *Y*-axis, dots) and RNA Integrity Number (RIN, right *Y*-axis, squares) of RNA isolated from six technical replicates across three independent experiments. Data are represented as mean ± SEM

To isolate nuclei from human islet preparations, we tested the Nuclei EZ lysis buffer-based protocol. We employed this isolation method because it was previously successfully used to isolate nuclei from frozen compact tissues, such as tumor tissues [58, 59]. In our hands, we validated that this protocol removes cytoplasmic content quickly and consistently. To test the efficacy of this protocol, we used a frozen sample of non-diabetic human islets (donor 1, Fig. 1A). Briefly, the archived human islet sample, consisting of 500 human islet equivalents (IEQs) was subjected to homogenization using the Nuclei EZ buffer. After a short incubation period, the cytoplasmic fraction was removed by centrifugation, and the pellet containing the nuclear fraction was washed to remove cytoplasmic contamination. The nuclei were filtered, counted, and tested in control assays including DAPI staining, protein, and RNA evaluation. To preclude confounding factors such as the quality of the isolation and the purity of the nuclear preparation we also obtained whole islet cell samples by dispersing a fresh sample of non-diabetic human islets, containing 500 IEQs (donor 2, Fig. 1A).

First, the quality of the isolation protocol was evaluated by visualizing the nuclei samples obtained from frozen human islets under phase-contrast microscopy and by assessing cell integrity in comparison to intact cell samples obtained by dissociating fresh human islets, collected by a separate donor, into single cells (whole cells), as previously described [60] (Fig. 1B). Phase-contrast images of isolated nuclei samples showed that the cytoplasm was depleted completely in all the cells, and the nucleus was intact (Fig. 1B). Whereas, as expected, the cytoplasm surrounding the DAPI-stained nucleus was easily distinguished in the dispersed single islet cells (Fig. 1B). Moreover, the lysis of the outer cell membranes in the samples processed for nuclei isolation was confirmed by assessing cytoplasmic (GAPDH) and nuclear (Lamin A/C) proteins in the samples collected during and after the isolation process (Fig. 1C). Western blot analysis of nuclei (Nuc), cytoplasmic fractions collected after the first (Cyt A) and the second (Cyt B) centrifuge steps, obtained by processing human islets from donor 1, and the whole cell lysate (WCL) sample, obtained by processing human islets from donor 2, showed that the nuclear isolation process efficiently removed the cytoplasm from nuclear samples, and preserved nuclear integrity, consistent with the DAPI staining (Fig. 1B). Indeed, we were able to isolate 8.32 ± 1.6 × 10^5^ nuclei from ~ 500 IEQs consistent with previous reports [61]. The amounts of nuclei were adequate to perform snRNA-seq—which required only 10,000 nuclei, as previously reported [48]—as well as for validation experiments such as qRT-PCR.

Total RNA was isolated from the nuclear samples, and the yield and integrity were evaluated using a Bioanalyzer [62] (Fig. 1D, E). We were able to isolate 3.3 ± 0.7 μg RNA from isolated nuclear samples (*n* = 6; three independent experiments). The RNA yield was in the expected range, considering that a mammalian cell contains 10–20 pg of total RNA of which 20–30% resides in the nucleus [63, 64]. RNA integrity evaluated by measuring two major peaks representing 18S and 28S rRNA revealed an average RNA integrity number (RIN) of 7.6 ± 0.4 which indicates high quality with minimal degradation [62]. Based on the efficacy of the tested protocol for obtaining pure high-quality nuclei from human islet samples, we decided to use this methodology for subsequent single-nucleus RNA-seq experiments.

### Side-by-side comparison of scRNA-seq and snRNA-seq methods in cultured human islets

**Fig. 2:**
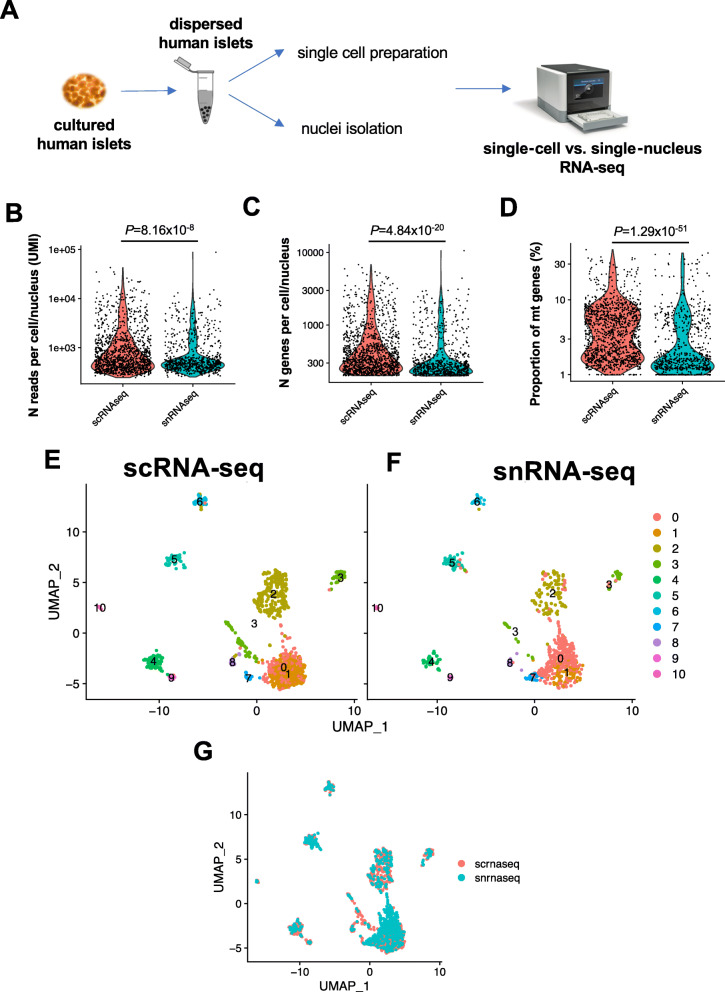
Side-by-side comparison of single-cell vs. single-nucleus RNA-sequencing in freshly cultured human islets. **A** Experimental design of the workflow for generating single-cell RNA-seq (scRNA-seq) and single-nucleus RNA-seq (snRNA-seq) datasets of cultured human islets. **B**–**D** Violin plots representing **B** number of reads (UMI), **C** number of genes, and **D** proportion (expressed in %) of mitochondrial genes per cell/nucleus in scRNA-seq (orange violins) and snRNA-seq (blue violins) datasets. Data are expressed in logarithmic scale (*Y*-axis). Statistical significance was tested using a Wilcoxon rank-sum test. **E**, **F** UMAP plots representing the clustering of the high-quality **E** cells and **F** nuclei in the **E** scRNA-seq and the **F** snRNA-seq datasets. *Y*-axis of plot in **F** is like the plot in **E**. **G** Global integrated UMAP plot representing the distribution of high-quality cells (from scRNA-seq, orange dots) and nuclei (from snRNA-seq, blue dots)

To compare the single-nucleus and single-cell RNA-sequencing procedures (snRNA-seq and scRNA-seq, respectively), we obtained human islets from a non-diabetic donor and divided them into two groups: (1) one to generate single-cell suspensions and (2) a second to isolate single nuclei using the protocol described above (Fig. 2A). We generated 4 technical replicates for each group of 10,000 cells or nuclei and loaded them into the 10X genomics Chromium Controller to obtain Gel-beads in Emulsion (GEMs). Following the 10X genomics standard protocol, we obtained single-cell and single-nuclei libraries that were then sequenced using next-generation sequencing (NGS). Following the application of the quality check filters, including removal of doublets and multiplets, we recovered 1277.2 ± 234.2 and 976.2 ± 82.0 (mean ± SD) high-quality cells and nuclei per sample, respectively (Additional file 2: Table S2). Although the number of reads and genes sequenced per cell/nucleus was slightly higher in the scRNA-seq compared to the snRNA-seq method (Fig. 2B, C), the duplication rate, indicating the ratio between usable vs. sequenced reads, as a read-out of sequencing efficiency, were similar between the two transcriptomic procedures (Additional file 3: Figure S1C, Additional file 2: Table S2). As confirmation of the high purity of the single-nucleus preparations, the percentage of mitochondrial genes sequenced in the nucleus-containing droplets was lower than the cell-containing particles (2.7% and 4.2%, respectively) (Fig. 2D, Additional file 2: Table S2). We obtained estimates of ambient RNA within the cell-/nucleus-containing droplets by applying Cell Bender [53], with averages of 5.87 ± 1.76 % and 1.42 ± 0.72 % (mean ± SD) among the four single-cell and four single-nucleus library replicates, respectively (Additional file 2: Table S2, Additional file 3: Figure S1A,B).

We clustered the data with Seurat, shown as UMAP plots of recovered cells (Fig. 2E) and nuclei (Fig. 2F). In particular, we were able to identify 10 clusters of cells and nuclei. Cells and nuclei expressing high levels of *INS* gene (natural log of counts > 3) were distributed in clusters #3, while *GCG*-enriched cells/nuclei were distributed in clusters #1 (Fig. 2E, F; Additional file 3: Figure S1D-G). Interestingly, cluster #7 was enriched in low *INS*-expressing nuclei and *GCG*-expressing nuclei, but not cells (Fig. 2E, F; Additional file 3: Figure S1D,E). Specimens expressing high levels of *SST* (natural log of counts > 3) were also found in clusters #3, while cells and nuclei expressing high levels of *PPY* (natural log of counts > 1.5) were distributed in clusters #1 and #3 (Fig. 2E, F’ Additional file 3: Figure S1H-K). In addition, cluster #0 in both the scRNA-seq and snRNA-seq sets comprised of cells and nuclei expressing low levels of all the endocrine cell marker genes (Fig. 2E, F; Additional file 3: Figure S1D-K). These findings suggest that the snRNA-seq protocol allows the identification of endocrine marker gene-expressing nuclei in a manner that is similar to scRNA-seq methods.

### Comparison of gene expression signatures of human islet cells using scRNA-seq and snRNA-seq

**Fig. 3:**
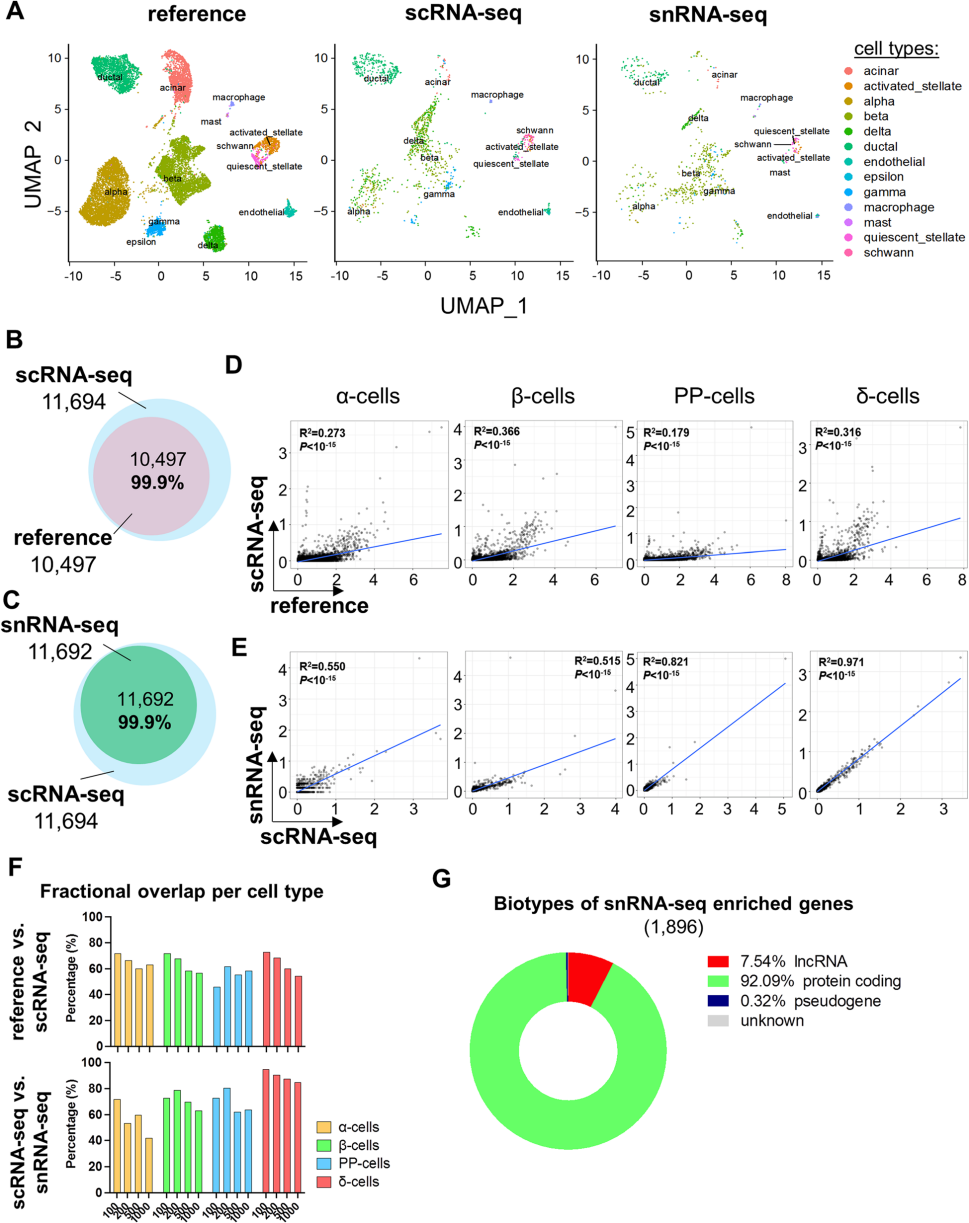
Comparison between snRNA-seq and scRNA-seq datasets following harmonization on reference dataset. **A** Global UMAP plots and cell type prediction in the scRNA-seq (middle panel) and snRNA-seq (right panel) following harmonization on the reference dataset generated by integrating five published scRNA-seq datasets of cultured human islets [21, 23, 26, 27, 57] (left panel). *Y*-axis is similar in all three panels. **B** Venn diagram of detected genes in the reference (pink circle) and in our scRNA-seq dataset (blue circle). **C** Venn diagram of detected genes in our scRNA-seq (blue circle) and in our snRNA-seq dataset (green circle). **D**, **E** Scatter plots of harmonized cell type-specific gene expression in α-cells, β-cells, PP-cells, or δ-cells **D** between the reference and the scRNA-seq datasets and **E** between the scRNA-seq and the snRNA-seq datasets. *X*-axis and *Y*-axis represent the expression levels in natural log of counts in the indicated datasets. The blue line in each plot represents the regression line, whose fit is indicated by the *R*^2^ value (the square of the Pearson correlation coefficient). The *P* and *R* values are provided for each correlation. **F** Fractional overlap expressed in percentages (%, *Y*-axis) of increasing numbers (100, 200, 500, and 1000, *X*-axis) of top genes within α-cells (yellow bars), β-cells (green bars), PP-cells (blue bars), or δ-cells (red bars) in reference vs. scRNA-seq (upper panel) and in scRNA-seq vs. snRNA-seq (lower panels) datasets following harmonization to the reference. **G** Pie chart representing proportions of biotypes of genes detected with higher confidence in snRNA-seq compared to scRNA-seq (snRNA-seq enriched genes)

To determine whether snRNA-seq would represent a reliable transcriptomic method to identify human islet cell types, we compared our snRNA-seq and scRNA-seq datasets with the publicly available scRNA-seq datasets. We chose five published scRNA-seq datasets of human pancreatic islets spanning 27 donors, four laboratories, and five technologies for harmonizing with Seurat [21,23,26,27,57]. We used this harmonized dataset as a “reference” (Fig. 3A) upon which to integrate our data, allowing for correspondence of cells and clusters. Indeed, when we compared the cultured human islet scRNA-seq and snRNA-seq datasets we had generated to the reference, we were able to identify all the major islet endocrine (i.e., α-cells, β-cells, PP-cells, δ-cells) and non-endocrine (endothelial cells, stellate cells, Schwann cells, acinar cells, and ductal cells) cell types previously reported using established scRNA-seq methodologies, suggesting that snRNA-seq protocols did not hinder the characterization of any islet cell type, including the low abundance PP-cell population (Fig. 3A, Additional file 4: Table S3). We then undertook one-to-one comparisons of global and cell type-specific gene expression profiles between (a) the scRNA-seq and the reference datasets and (b) between the scRNA-seq and the snRNA-seq datasets we had generated. We observed an almost total overlap (99.9 %) of the genes detected between the reference (10,497 genes) and our scRNA-seq (11,694 genes) and between the scRNA-seq and the snRNA-seq (11,692 genes) datasets (Fig. 3B, C). We tested the association of gene expression levels between datasets within the major islet cell types (α-cells, β-cells, PP-cells, and δ-cells) and observed positive correlations within α-cells, β-cells, PP-cells, and δ-cells between scRNA-seq and the reference (Fig. 3D). In addition, such correlations were driven by cell type-specific genes, such as *GCG* for α-cells, *INS* for β-cells, and so forth, an aspect that was absent in all the other correlations between a given islet cell type from the scRNA-seq dataset and a different one from the reference output (Additional file 3: Figure S2A). Notably, the positive correlations of the gene signatures were also evident within α-cells, β-cells, PP-cells, and δ-cells in the scRNA-seq and snRNA-seq dataset comparisons (Fig. 3E).

We then analyzed the fractional overlap of increasing *N* number of top genes (*N* = 100, 200, 500, and 1000 top genes) within cell types between the scRNA-seq and the reference and between the snRNA-seq and the scRNA-seq datasets (Fig. 3F). In particular, a significant proportion (∼ 72%) of the top 100 transcripts was shared by the major islet cell types (α-cells, β-cells, and δ-cells) between the reference and the scRNA-seq datasets (Fig. 3F, Additional file 3: Figure S2B-E). Similar levels of fractional overlap of an increasing number of top genes were also observed in the scRNA-seq vs. snRNA-seq comparison (Fig. 3F). Curiously, the δ-cell groups displayed the highest overlap, reaching 95% in the top 100 and 85% in the top 1000 cell type genes (Fig. 3F). These results support the notion that snRNA-seq identifies genes expressed in the least as well as the most abundant endocrine cells, providing confidence that snRNA-seq is a reliable approach for identifying genes expressed in the various islet cell types.

To evaluate whether snRNA-seq would allow identification of specific types of genes which are covered with higher confidence compared to scRNA-seq, we analyzed the biotypes of the genes detected in the snRNA-seq dataset with a significantly higher (> 1.5-folds) percentage of detection compared to the scRNA-seq dataset. Among the 1896 genes which were covered in the snRNA-seq with a higher confidence compared to the scRNA-seq method, 7.5% accounted for long non-coding RNAs (lncRNAs), whereas 92.1% were protein coding genes (Fig. 3G). Importantly, lncRNA genes were also among the top genes detected in snRNA-seq with higher confidence compared to scRNA-seq protocols. Indeed, the proportions of detection of cytochrome c oxidase assembly factor heme A:farnesyltransferase (COX10) antisense RNA 1 (*COX10-AS1*) and minichromosome maintenance complex component 3 associated protein (MCM3AP) antisense RNA 1 (*MCM3AP-AS1*) genes in snRNA-seq were 15.5-fold and 13.7-fold higher than scRNA-seq, respectively (Additional file 5). In addition, nuclear paraspeckle assembly transcript 1 (*NEAT1*) and maternally expressed 3 (*MEG3*), also lncRNA genes, were detected in the snRNA-seq dataset with a proportion of 70% and 26.9%, respectively, whereas their detection rates in the scRNA-seq dataset were 35.5% and 7.0%, respectively (Additional file 5). These data suggested that the snRNA-seq method allows for detecting nuclei-enriched lncRNA genes with a higher confidence compared to scRNA-seq. The ability of the nuclear transcriptomic analysis to detect genes enriched in non-coding RNAs provides an important resource for studying the epigenetic regulatory mechanisms in human islets.

### snRNA-seq of transplanted human islets

**Fig. 4:**
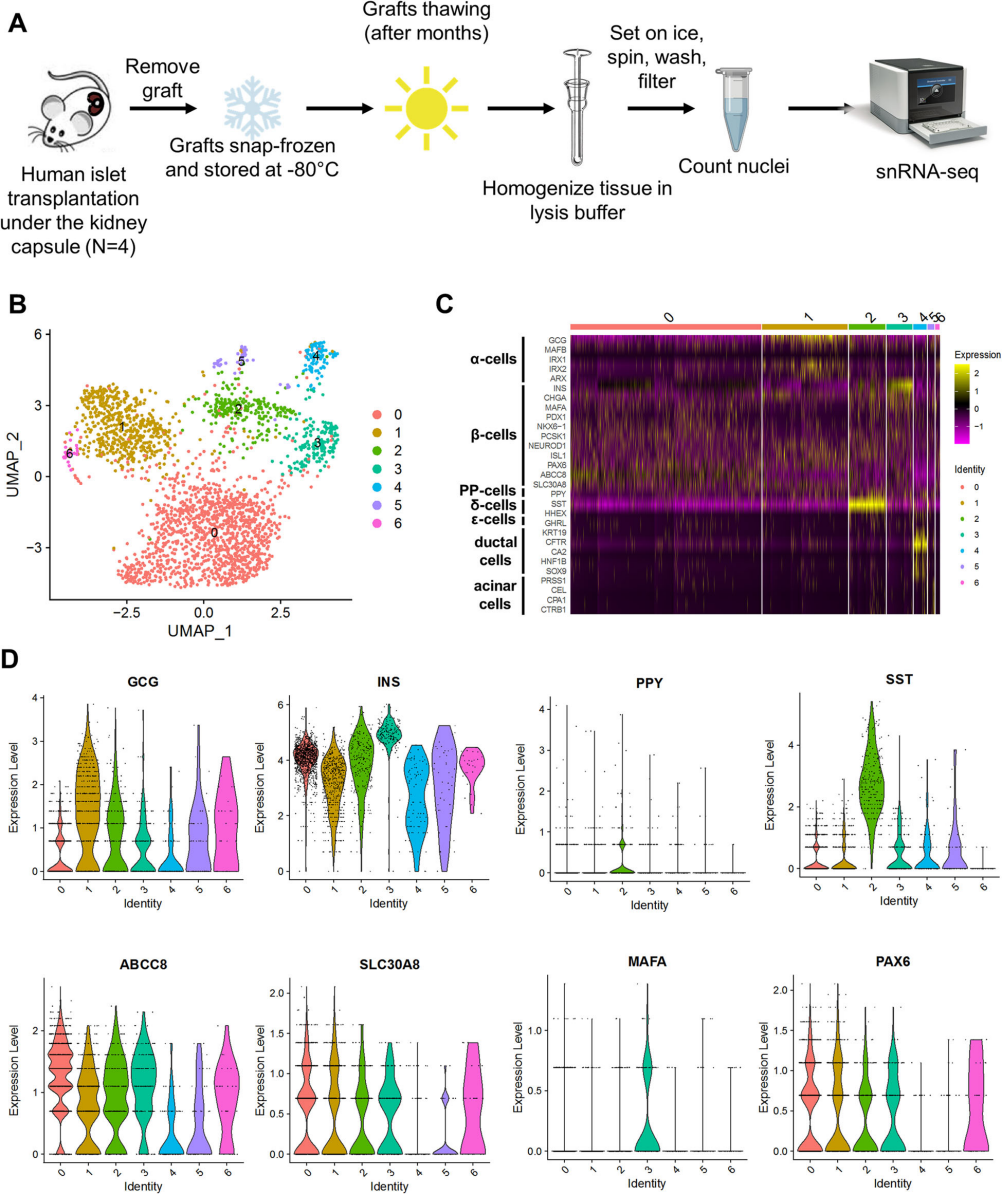
Single-nucleus RNA-sequencing in human islet grafts. **A** Experimental scheme of isolation of nuclei and snRNA-seq protocol in transplanted human islets. **B** Global UMAP plot displaying clustering of high-quality nuclei derived from engrafted human islets. **C** Heatmap representing standardized gene expression (standardized natural log of counts) of selected endocrine and exocrine gene markers in each nucleus separated by cluster. Each column represents a single nucleus, and each row is a different gene. (**D**, upper panels) Violin plots representing the expression levels of endocrine cell gene markers, including insulin (*INS*), glucagon (*GCG*), pancreatic polypeptide (*PPY*), or somatostatin (*SST*) in each nuclear cluster. (**D**, lower panels) Violin plots representing the expression levels of β-cell gene markers, such as *ABCC8*, *SLC30A8*, *MAFA*, and endocrine markers, such as *PAX6* within the nuclear clusters. Expression levels (*Y*-axis) are natural log of counts

To reveal the transcriptomic signature of frozen engrafted human islets, we undertook snRNA-seq experiments as depicted in Fig. 4A. We used the immunodeficient NSG mouse model which is a widely utilized in vivo model for β-cell regeneration studies [6, 8, 10, 65]. Human islets (1000 IEQs) obtained from 4 different donors were transplanted individually under the kidney capsule of 8-to-12-week-old male mice and followed up for 4 weeks. At the end of 4 weeks, grafts were removed carefully and divided into two equivalent parts (~ 500 IEQs each) that were subsequently snap frozen. On the day of nuclei isolation, frozen graft fractions (~ 500 IEQs) were placed in ice-cold lysis buffer individually and homogenized immediately to obtain pure nuclei as described above (Fig. 1) and single-nucleus cDNA libraries were generated by using the Chromium Single-Cell 3’ Library Kit (Fig. 4A) [41]. Similar to the analyses of cultured islet snRNA-seq, the engrafted islet snRNA-seq data were initially analyzed using quality check pipelines in order to remove low-quality nuclei, including those with a total number of reads (UMI) < 1000, a total number of expressed genes < 500, and a proportion of mitochondrial genes > 20%, in line with previous reports [35, 36, 38] (Additional file 2: Table S2, Additional file 3: Figure S3A-C). It is worth noting that the percentage of expression of mitochondrial genes per nucleus was < 2% of the whole transcriptome (Additional file 2: Figure S3C), in line with the in vitro snRNA-seq results, suggesting a high efficiency of nuclear isolation method and allowing for a greater confidence to interpret and analyze the snRNA-seq data [36]. In addition, by aligning the recovered reads to the murine genome (GRCm38), we filtered out nuclei containing > 25% mouse-specific UMI (Additional file 3: Figure S3D). Finally, Cell Bender estimated ambient RNA contamination levels at 4.6 ± 1.3 % (Additional file 2: Table S2; Additional file 3: Figure S3E) within nucleus-containing droplets. With this approach, we recovered 3565 (891.2 ± 409.6 per sample) high-quality nuclei, which is comparable with previous studies using a similar [35] or different platforms [35, 42, 66, 67].

Next, clustering with Seurat yielded 7 major clusters (Fig. 4B). Notably, we observed a cluster of nuclei enriched in α-cell-specific genes, namely cluster 1; a nuclear cluster enriched in *SST* and *PPY* genes, namely cluster 2; and a high *INS* and MAF bZIP transcription factor A (*MAFA)*-expressing nuclear cluster (natural log of counts ≥ 5), namely cluster 3 (Fig. 4B–D). Interestingly, other β-cell marker genes, including ATP binding cassette subfamily C member 8 (*ABCC8*) and solute carrier family 30 member 8 (*SLC30A8*), were also expressed in cluster 0, which displayed low levels of *INS* expression (natural log of counts < 5) (Fig. 4B–D). In addition, cluster 0 was enriched in endocrine cell-specific genes, such as paired box 6 (*PAX6*), suggesting that such clusters likely included β-cells in a different maturity state. However, low *INS* expression levels were also found in nuclei belonging to cluster 2 (Fig. 4B–D). Regarding the exocrine cell marker gene expression, a cluster of nuclei enriched in ductal cell genes—including CF transmembrane conductance regulator (*CFTR)*, SRY-box transcription factor 9 (*SOX9*), and keratin 19 (*KRT19*), was identified in cluster 4, whereas the acinar cell-specific genes were virtually absent (Fig. 4B,C). Finally, we harmonized the in vivo snRNA-seq to the reference dataset to evaluate an unsupervised prediction of the cell type identity. Strikingly, all the endocrine and non-endocrine islet cell types were identified following harmonization, indicating the efficacy of the snRNA-seq approach to recover virtually all islet cells from transplanted samples (Additional file 4: Table S3, Additional file 3: Figure S3F). Taken together, these data indicate that snRNA-seq of human islet grafts reveals the presence of genes marking all islet cell types, and as expected, a virtual absence of exocrine cell genes.

The presence of nuclei with high *SST*-expression and low *INS*-expression in cluster 2 prompted us to undertake immunofluorescence studies to validate polyhormonal expression at the protein level. To this end, we immunostained α-cells, β-cells, and δ-cells in graft sections following transplantation of human islets from the same donors used for the in vivo transcriptomic analysis. We observed overlap between SST and INS protein immunostaining in transplanted human islets, suggesting that, indeed, cell fate transition occurs over the 4-week in vivo engraftment period, as previously observed [51, 52, 68] (Additional file 3: Figure S4).

### Comparison of in vitro vs. in vivo single-nucleus RNA-sequencing methods

**Fig. 5:**
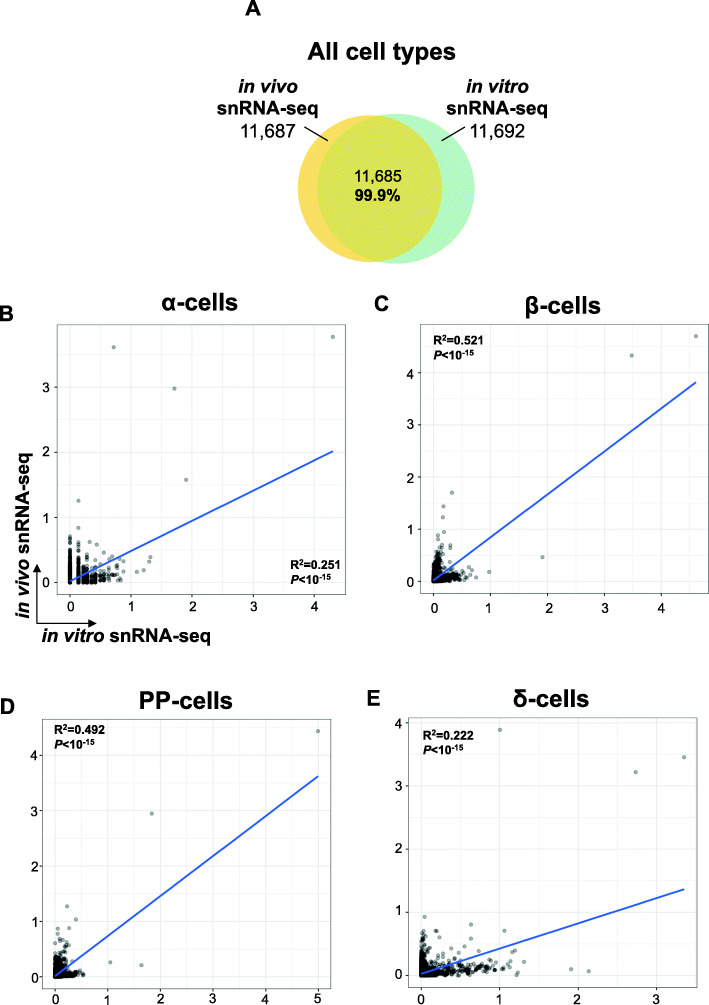
Comparison between in vitro and in vivo snRNA-seq datasets. **A** Intersection of the total number of detected genes in the snRNA-seq dataset of cultured human islets (in vitro snRNA-seq, green circle) and the snRNA-seq dataset of transplanted human islets (in vivo snRNA-seq, yellow circle). **B**–**D** Scatter plots of harmonized cell type-specific gene expression in **B** α-cells, **C** β-cells, **D** PP-cells, and **E** δ-cells between the in vitro and the in vivo snRNA-seq datasets. *X*-axis and *Y*-axis represent the gene expression levels expressed as natural log of counts in the indicated datasets. The blue line in each plot represents the regression line, whose fit is indicated by the *R*^2^ value (the square of the Pearson correlation coefficient). The *P* and *R* values are provided for each correlation

To determine the reliability of the snRNA-seq dataset generated from transplanted human islets, we compared the transcriptomic profiles of the snRNA-seq output obtained from cultured (in vitro) with that from transplanted (in vivo) human islets. The genes detected in each dataset showed a significant overlap (99.9%; diagram Fig. 5A). Testing the association of the islet cell type gene expression profiles revealed a positive correlation in α-cells and δ-cells (Fig. 5B, E) that was stronger in β-cells and PP-cells between in vitro and the in vivo snRNA-seq datasets (Fig. 5C, D). These data confirm the similarity of transcriptomic profiles between freshly cultured and frozen engrafted human islets and highlight the ability of snRNA-seq procedures to interrogate gene expression of human islet cells.

## Discussion

Over the last few decades, the research community is increasingly utilizing human islet studies with the long-term goal of developing novel translational strategies to counteract diabetes [69]. Moreover, the establishment of avant-garde technologies for studying biological processes at the single-cell resolution have provided new tools for exploring human islet cells in physiological conditions and their defects during diabetes disease progression [25–28, 70]. While such studies were commonly conducted on cultured human islets in vitro, the transcriptome of frozen human islet cells after transplantation in mouse models remains largely unexplored and provides an opportunity to study archived tissues. Analyzing the transcriptomic signatures of human islet cells at the single-cell level represents a state-of-the-art tool for gathering insights into dynamic molecular mechanism(s) in response to diverse stimuli, including mitogens, differentiating factors, or stimulators of hormone secretion. In this context, human β-cell proliferation, differentiation/transdifferentiation, or neogenesis have all been topics of investigation in the context of physiological (e.g., pregnancy) or pathophysiological (e.g., insulin resistance/T2D) states. Nevertheless, there continues to be an urgent need for new tools to reliably study archived human or mouse tissues. Here we present single-nucleus RNA-sequencing (snRNA-seq) for interrogating the human islet transcriptome that is especially relevant for small samples that have been frozen following in vivo manipulation.

It has been established that the isolation of nuclei has several advantages over single-cell isolation [35, 67, 71]. First, this method can be simultaneously applied to multiple samples that are collected at different time points, reducing the potential variations introduced by sample handling. The second advantage is that the nucleus isolation method is efficient and requires fewer steps compared to the single-cell protocol. For example, snRNA-seq does not require enzymatic digestion to dissociate tissues into single cells which often results in decreased viability and cell loss [72]. Finally, rapid isolation of nuclei compared to tissue dissociation and isolation of single cells minimizes changes in the transcriptome during the isolation process [31]. Such considerations are important, especially when the volume of tissue available for studies is necessarily limited, such as human islet grafts.

To test the reliability of snRNA-seq in comparison to the well-established scRNA-seq procedures, we undertook a direct comparison between the two methodologies by analyzing the transcriptomics of freshly isolated cells and nuclei obtained from the same human islet donor. Despite the differences in terms of sequenced reads and detected genes per specimen between the two sequencing procedures, likely due to the lower RNA content in the nucleus compared to the cytosol [63], the sequencing efficiency was similar between scRNA-seq and snRNA-seq procedures, consistent with reports on other metabolic tissues [35, 67, 71]. We also generated a reference dataset by harmonizing 5 previously published scRNA-seq datasets in human islets in order to compare our transcriptomic results in an unsupervised fashion [56]. This approach indicated that snRNA-seq allowed for the identification of all the pancreatic islet cell types, including the least abundant, such as the PP-cells. The data also showed an almost total overlap in global gene expression (99.9%) between the two methodologies highlighted by positive correlations of gene signatures, mainly driven by cell-specific genes, between each of the islet cell types. Furthermore, the ability to identify genes whose percentage of detection in snRNA-seq was > 1.5-fold higher than scRNA-seq indicated those genes were detected by snRNA-seq with a higher confidence in comparison to scRNA-seq. The fact that ~ 7.5% of the snRNA-seq enriched genes were lncRNAs suggested that snRNA-seq represented a potential tool for identifying non-coding RNAs in human islets that would be useful to examine chromatin remodeling, post-transcriptional modifications, and crosstalk with other RNA species.

We then applied snRNA-seq to archived human islet graft samples. As demonstrated for the cultured islet samples, single-nucleus preparations from transplanted human islets allowed the identification of all the islet cell types with a coverage that is comparable to single-cell profiling. Notably, the global and the islet cell-specific single-nucleus transcriptomics of cultured versus transplanted human islet were highly concordant, confirming that snRNA-seq represents a reliable strategy to analyze the gene signature of human islets in vivo at single-cell resolution. Finally, the transcriptome of polyhormonal islet cell clusters, e.g., cluster 2 expressing *INS* and *SST*, could be recapitulated at the protein level by immunofluorescence. Although other in situ hybridization methods, such as RNA-scope, would be more appropriate to validate transcriptomic results, the validation at the protein level provides an important functional perspective to the snRNA-seq data.

## Conclusions

We propose snRNA-seq as a reliable tool to explore the transcriptomic profile of human islets especially from frozen archived samples which may not be ideal for single-cell procedures.

## Supplementary Information

**Additional file 1: Table S1.** Characteristics of human islet donors and human islet preparations used for single-cell or single-nucleus transcriptomic analysis.

**Additional file 2: Table S2.** Number of different types of droplets, reads and genes generated by scRNA-seq and snRNA-seq in cultured or transplanted human islets.

**Additional file 3: Figure S1.** Ambient contribution and islet cell gene marker UMAP and violin plots in scRNA-seq and snRNA-seq in cultured human islets. (A,B) Box plots representing the levels of ambient RNA contamination in droplets of (A) scRNA-seq and (B) snRNA-seq datasets. (C) Violin plot representing the duplication rate in scRNA-seq (orange plot) and snRNA-seq (blue plot) methodologies. The rate was calculated by normalizing the average number of UMI per cell/nucleus on the average number of reads per cell/nucleus. (D, F, H, J) UMAP plots displaying expression levels of (D) *GCG*, (F) *INS*, (H) *PPY*, and (J) *SST* within the global distribution in scRNA-seq (left panels) and snRNA-seq (right panels). Expression levels are indicated as natural log of counts and range from 0 (gray) to 5 (purple). (E, G, I, K) Violin plots representing expression levels (natural log of counts, *Y*-axis) of (E) *GCG*, (G) *INS*, (I) *PPY*, and (K) *SST* within each cluster (*X*-axis) identified in scRNA-seq (orange plots) or snRNA-seq (blue plots) datasets. **Figure S2.** Correlation plots and fractional overlap estimation of islet cell types from scRNA-seq dataset with those from the reference dataset. (A) Scatter plots representing correlation of gene expression levels between scRNA-seq-derived (*Y*-axis) α-cells (first row from top), β-cells (second row from top), PP-cells (third row from top), and δ-cells (fourth row from top) and reference-derived (*X*-axis) α-cells (first column from left), β-cells (second column from left), PP-cells (third column from left) or δ-cells (fourth column from left). X-axis and Y-axis represent the expression levels in natural log of counts in the indicated datasets. The blue line in each plot represents the regression line, whose fit is indicated by the R^2^ value (the square of the Pearson correlation coefficient). The red circles indicate the islet cell specific marker genes driving the correlation between the same cell type from the two datasets. The red dotted squares highlight the correlation plots used in the main Fig. 3D. (B-E) Fractional overlap expressed in percentages (%, *Y*-axis) of the (B) top 100, (C) 200, (D) 500, or (E) 1000 genes between the indicated islet cell types from the reference dataset (*X*-axis) and the α-cells (yellow bars), β-cells (green bars), PP-cells (blue bars), or δ-cells (red bars) from the scRNA-seq dataset. **Figure S3.** Quality check parameters in the transplanted human islet snRNA-seq dataset. (A-D) Violin plots of (A) number of genes, (B) number of reads, (C) proportion of mitochondrial genes, and (D) proportion of mouse genes per nucleus across the 4 transplanted human islet samples in the snRNA-seq dataset. (E) Box plot of the levels of ambient RNA contamination in droplets of the in vivo snRNA-seq dataset within each human islet graft. (F) UMAP plot of nuclear cluster distribution and cell type prediction of in vivo snRNA-seq dataset following harmonization to the reference dataset. **Figure S4.** Islet cell type validation in human islet graft sections by immunofluorescence. Representative images of human islet cells identified as α-cells, β-cells or δ-cells according to the glucagon (GCG, green), insulin (INS, red) and somatostatin (SST, white) labeling. Nuclei are stained in blue. Scale bar is: 50 μm.

**Additional file 4: Table S3.** Number of endocrine and exocrine pancreatic cell types predicted in scRNA-seq or snRNA-seq of cultured or transplanted human islets following harmonization.

**Additional file 5.** List of top 20 snRNA-seq enriched genes ordered by fold change of percentage of detection (upper table) or percentage of detection (lower table) compared to scRNA-seq.

## Data Availability

All raw and processed snRNA-seq data generated from engrafted human islet tissues have been submitted to the NCBI Gene Expression Omnibus (GEO; https://www.ncbi.nlm.nih.gov/geo/query/acc.cgi?acc=GSE150212) under accession number GSE150212. The publicly available scRNA-seq datasets on human islets that were used in this the manuscript to integrate our scRNA-seq and snRNA-seq datasets have been deposited in the public Genomic Spatial Event Database (GSE) and in the ArrayExpress Database at the EMBL-EBI. For InDrop, accession number GSE84133 (https://www.ncbi.nlm.nih.gov/geo/query/acc.cgi?acc=GSE84133) [21]; for CelSeq2, accession number GSE85241 (https://www.ncbi.nlm.nih.gov/geo/query/acc.cgi?acc=GSE85241) [23]; for SMART-Seq2, accession number E-MTAB-5061 (https://www.ebi.ac.uk/arrayexpress/experiments/E-MTAB-5061/) [26]; for Fluidigm C1, accession number GSE86469 (https://www.ncbi.nlm.nih.gov/geo/query/acc.cgi?acc=GSE86469) (27); and for CelSeq, accession number GSE81076 (https://www.ncbi.nlm.nih.gov/geo/query/acc.cgi?acc=GSE81076) [57] were used.
