# Supplementary material for: Using single-nucleus RNA-sequencing to interrogate transcriptomic profiles of archived human pancreatic islets
Source: Genome Med. 2021 Aug 10;13:128. doi: 10.1186/s13073-021-00941-8 (PMC8356387; doi:10.1186/s13073-021-00941-8)
Supplement: Supplementary file 1 — Additional file 1: Table S1. Characteristics of human islet donors and human islet preparations used for single-cell or single-nucleus transcriptomic analysis. [file 13073_2021_941_MOESM1_ESM.docx]

| **Application** | **UNOS ID** | **Gender (%)** | **Age range (years)** | **Height average (cm)** | **Weight average (kg)** | **BMI average (kg/m^2^)** | **HbA1C average (%)** | **Ethnicity (%)** | **Cause of death (%)** | **Viability average (%)** | **Purity**  **average (%)** |
| --- | --- | --- | --- | --- | --- | --- | --- | --- | --- | --- | --- |
| *in vitro* scRNA-seq vs snRNA-seq comparison | HP-21006-01 | M:40 F: 60 | 37-57 | 169.2 | 77.2 | 26.2 | 5.72 | Asian: 40  Hispanic: 20  Caucasian: 40 | Stroke: 40  Anoxia: 60 | 95 | 90 |
| *in vivo* snRNA-seq | ADJP050 |  |  |  |  |  |  |  |  |  |  |
|  | AEGN337 |  |  |  |  |  |  |  |  |  |  |
|  | AEGI394 |  |  |  |  |  |  |  |  |  |  |
|  | AEIR167 |  |  |  |  |  |  |  |  |  |  |

**Table S1: Characteristics of human islet donors and human islet preparations.**

BMI: Body Mass Index, HbA1C: glycated hemoglobin
