# Supplementary material for: Using single-nucleus RNA-sequencing to interrogate transcriptomic profiles of archived human pancreatic islets
Source: Genome Med. 2021 Aug 10;13:128. doi: 10.1186/s13073-021-00941-8 (PMC8356387; doi:10.1186/s13073-021-00941-8)
Supplement: Supplementary file 2 — Additional file 2: Table S2. Number of different types of droplets, reads and genes generated by scRNA-seq and snRNA-seq in cultured or transplanted human islets. [file 13073_2021_941_MOESM2_ESM.docx]

| **Sample** | **Dataset** | **Sample ID** | **Empty droplets** | **Doublets** | **Single cell/nucleus containing droplets** | **Reads per cell/nucleus (UMI)** | **Genes per cell/nucleus** | **Ambient RNA (%)** | **Mitochondrial genes  (%)** | **Murine genes (%)** |
| --- | --- | --- | --- | --- | --- | --- | --- | --- | --- | --- |
| cultured human islets | scRNA-seq | SC1 | 736110 | 40 | 1123 | 1506.9 | 582.8 | 3.7 | 4.4 | N/A |
|  |  | SC2 | 735578 | 63 | 1625 | 1018.3 | 441 | 8 | 4.1 | N/A |
|  |  | SC3 | 736076 | 38 | 1157 | 1648.9 | 619.3 | 6.1 | 4.2 | N/A |
|  |  | SC4 | 736022 | 44 | 1204 | 2024.8 | 689.7 | 5.7 | 4.1 | N/A |
|  | snRNA-seq | SN1 | 736312 | 32 | 936 | 1178.8 | 499.3 | 2.5 | 3.1 | N/A |
|  |  | SN2 | 736149 | 33 | 1091 | 3058.8 | 786 | 1.0 | 3.6 | N/A |
|  |  | SN3 | 736343 | 29 | 903 | 920.1 | 404.4 | 1.1 | 2.1 | N/A |
|  |  | SN4 | 736268 | 30 | 975 | 819.3 | 368.9 | 1.1 | 2 | N/A |
| transplanted human islets | snRNA-seq | SK01 | 736896 | 11 | 373 | 1630.9 | 985.9 | 4.4 | 0.2 | 15 |
|  |  | SK02 | 736129 | 44 | 1106 | 1340.4 | 744 | 2.8 | 0.4 | 29.4 |
|  |  | SK09 | 736471 | 30 | 776 | 1802.1 | 1100.2 | 5.4 | 0.2 | 45.7 |
|  |  | SK10 | 735913 | 51 | 1310 | 1176.4 | 705.6 | 5.8 | 0.2 | 16 |

**Table S2: Number of different types of droplet, reads and genes generated by scRNA-seq and snRNA-seq in cultured or transplanted human islets.**
