# Supplementary material for: Using single-nucleus RNA-sequencing to interrogate transcriptomic profiles of archived human pancreatic islets
Source: Genome Med. 2021 Aug 10;13:128. doi: 10.1186/s13073-021-00941-8 (PMC8356387; doi:10.1186/s13073-021-00941-8)

Basile G. Fig. S1

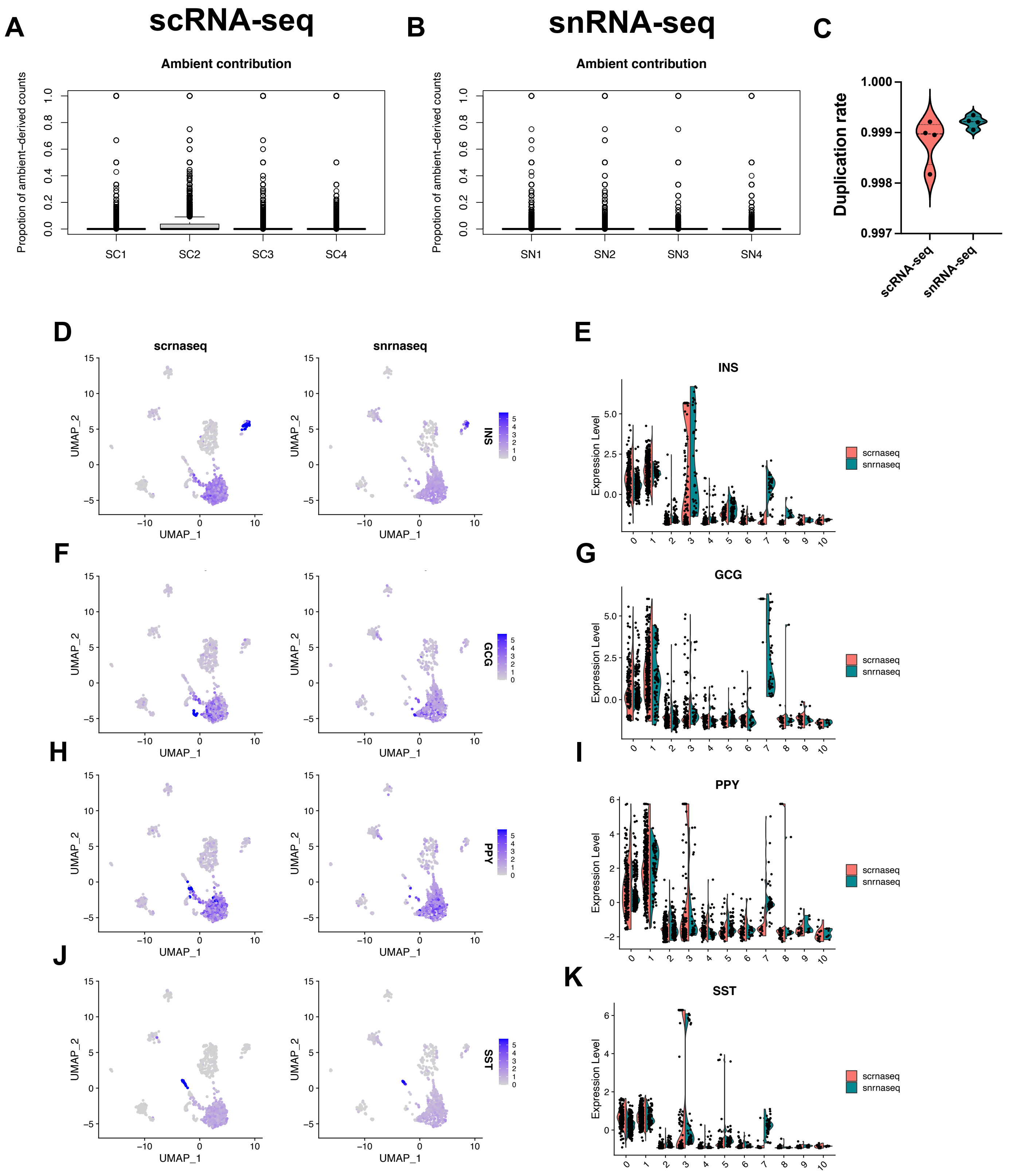

Basile G. Fig. S2

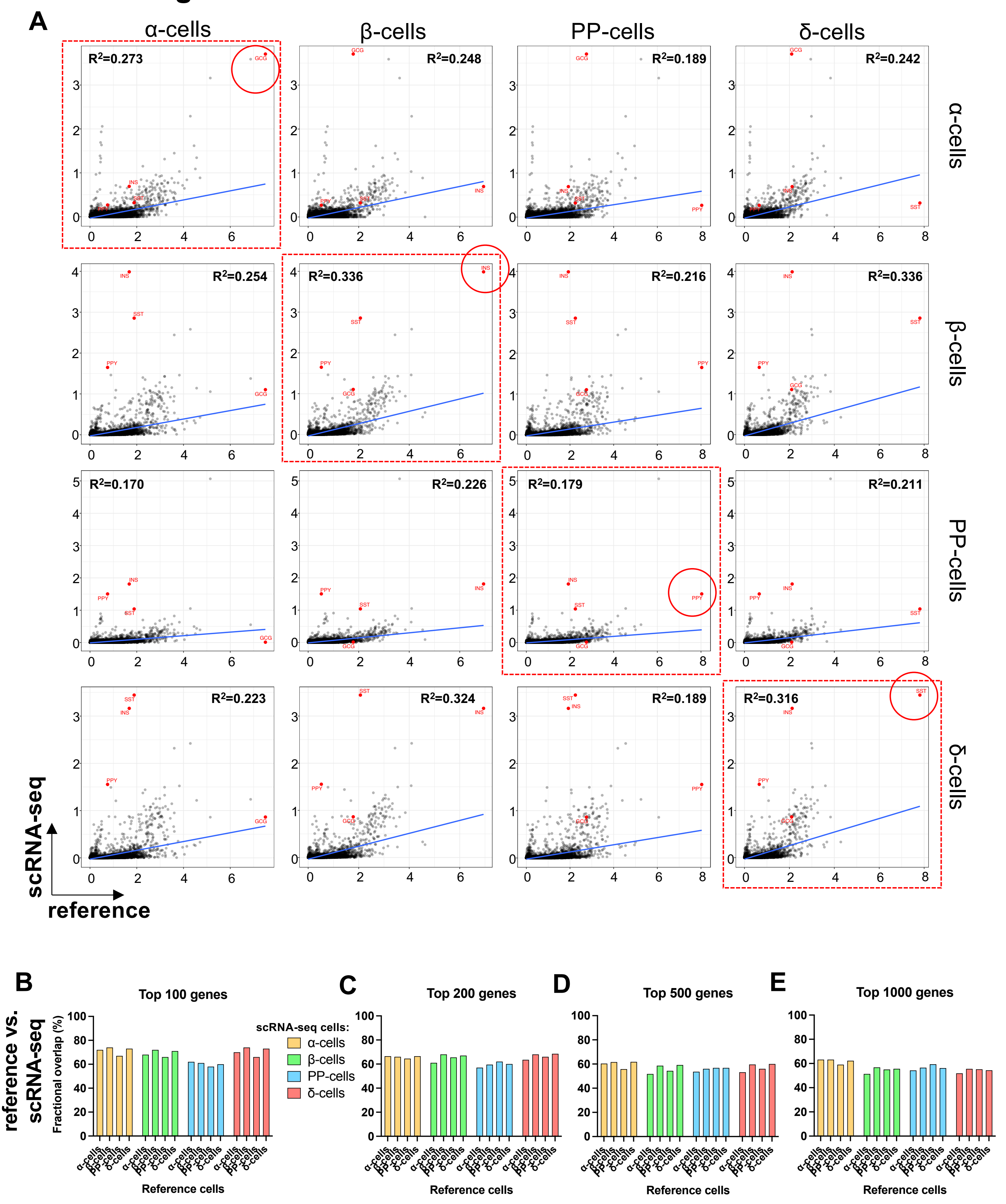

Basile G. Fig. S3

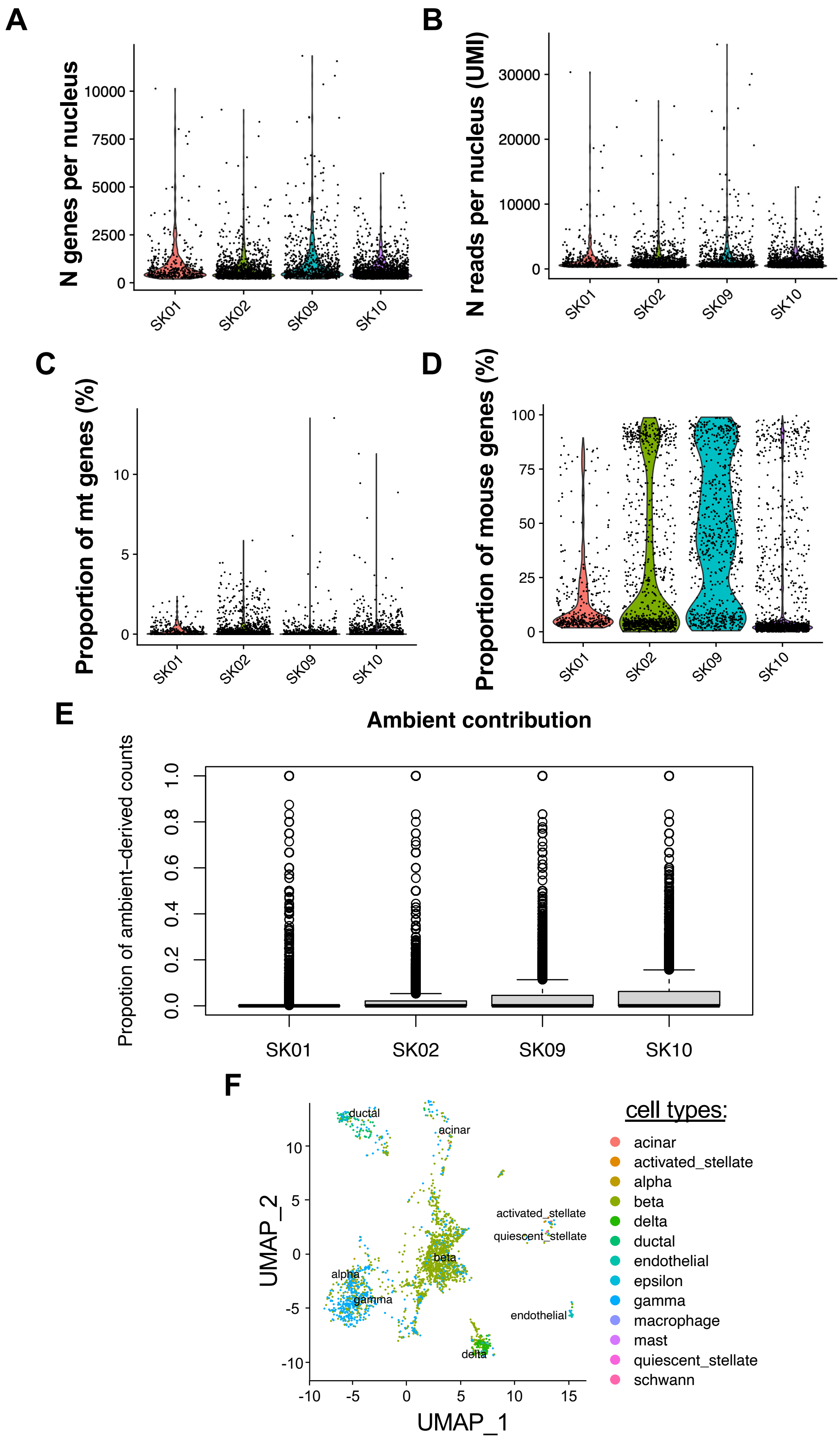

Basile G. Fig. S4

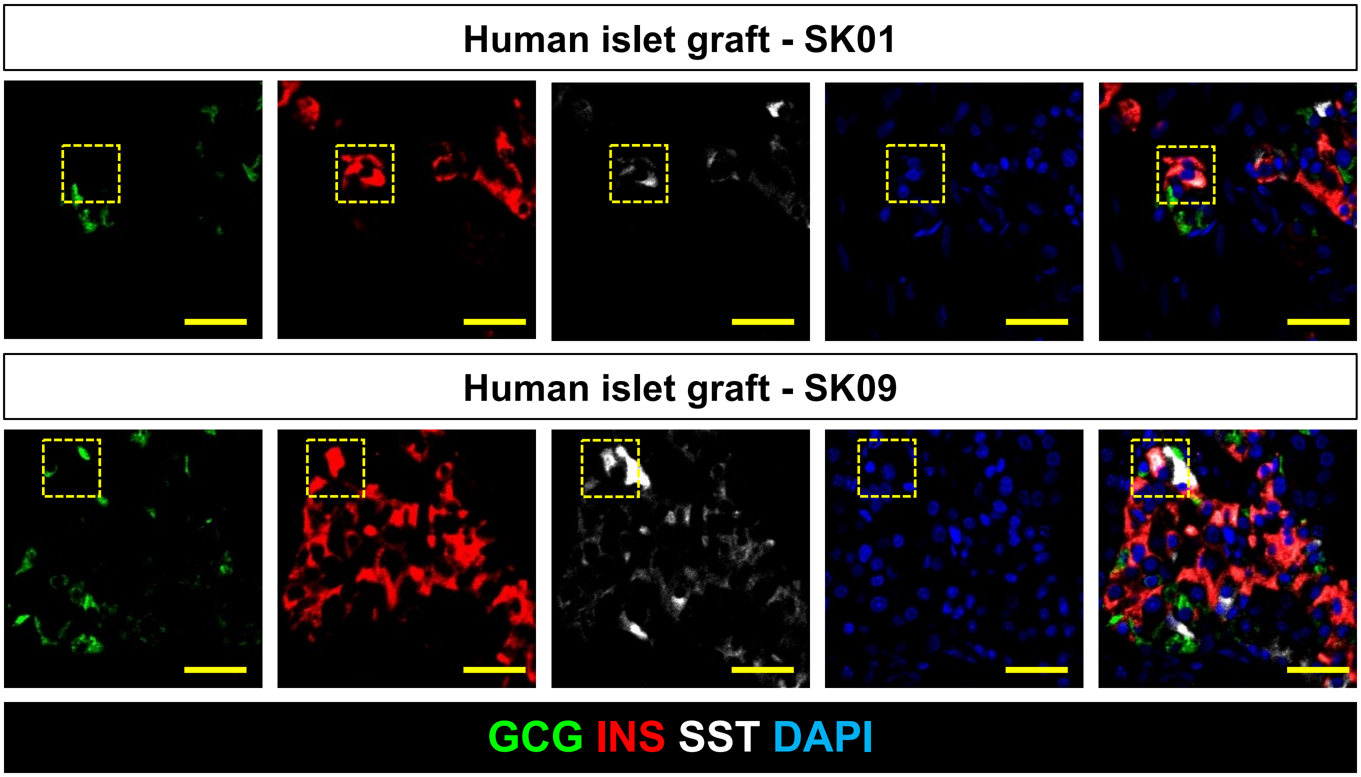

Supplement: Supplementary file 3 — Additional file 3: Figure S1. Ambient contribution and islet cell gene marker UMAP and violin plots in scRNA-seq and snRNA-seq in cultured human islets. (A,B) Box plots representing the levels of ambient RNA contamination in droplets of (A) scRNA-seq and (B) snRNA-seq datasets. (C) Violin plot representing the duplication rate in scRNA-seq (orange plot) and snRNA-seq (blue plot) methodologies. The rate was calculated by normalizing the average number of UMI per cell/nucleus on the average number of reads per cell/nucleus. (D, F, H, J) UMAP plots displaying expression levels of (D) GCG, (F) INS, (H) PPY, and (J) SST within the global distribution in scRNA-seq (left panels) and snRNA-seq (right panels). Expression levels are indicated as natural log of counts and range from 0 (gray) to 5 (purple). (E, G, I, K) Violin plots representing expression levels (natural log of counts, Y-axis) of (E) GCG, (G) INS, (I) PPY, and (K) SST within each cluster (X-axis) identified in scRNA-seq (orange plots) or snRNA-seq (blue plots) datasets. Figure S2. Correlation plots and fractional overlap estimation of islet cell types from scRNA-seq dataset with those from the reference dataset. (A) Scatter plots representing correlation of gene expression levels between scRNA-seq-derived (Y-axis) α-cells (first row from top), β-cells (second row from top), PP-cells (third row from top), and δ-cells (fourth row from top) and reference-derived (X-axis) α-cells (first column from left), β-cells (second column from left), PP-cells (third column from left) or δ-cells (fourth column from left). X-axis and Y-axis represent the expression levels in natural log of counts in the indicated datasets. The blue line in each plot represents the regression line, whose fit is indicated by the R2 value (the square of the Pearson correlation coefficient). The red circles indicate the islet cell specific marker genes driving the correlation between the same cell type from the two datasets. The red dotted squares [file 13073_2021_941_MOESM3_ESM.pdf]
