# Supplementary material for: Using single-nucleus RNA-sequencing to interrogate transcriptomic profiles of archived human pancreatic islets
Source: Genome Med. 2021 Aug 10;13:128. doi: 10.1186/s13073-021-00941-8 (PMC8356387; doi:10.1186/s13073-021-00941-8)
Supplement: Supplementary file 4 — Additional file 4: Table S3. Number of endocrine and exocrine pancreatic cell types predicted in scRNA-seq or snRNA-seq of cultured or transplanted human islets following harmonization. [file 13073_2021_941_MOESM4_ESM.docx]

| **Sample** | **Dataset** | **α-cells** | **β-cells** | **PP-cells** | **δ-cells** | **acinar-cells** | **ductal-cells** |
| --- | --- | --- | --- | --- | --- | --- | --- |
| cultured human islets | reference | 4615 | 3679 | 625 | 1013 | 1854 | 1954 |
|  | scRNA-seq | 16 | 355 | 62 | 271 | 23 | 360 |
|  | snRNA-seq | 5 | 414 | 42 | 67 | 10 | 106 |
| transplanted human islets | snRNA-seq | SK0122 | 1468 | 698 | 103 | 2 | 56 |

**Table S3: Number of endocrine and exocrine pancreatic cell types predicted in scRNA-seq or snRNA-seq of cultured or transplanted human islets following harmonization**
